# Remote Sensing Image Dehazing through an Unsupervised Generative Adversarial Network

**DOI:** 10.3390/s23177484

**Published:** 2023-08-28

**Authors:** Liquan Zhao, Yanjiang Yin, Tie Zhong, Yanfei Jia

**Affiliations:** 1 Key Laboratory of Modern Power System Simulation and Control & Renewable Energy Technology, Ministry of Education, Northeast Electric Power University, Jilin 132012, China; 2202100363@neepu.edu.cn (Y.Y.); zht@neepu.edu.cn (T.Z.); 2 College of Electric and Information Engineering, Beihua University, Jilin 132021, China; jiayanfei@beihua.edu.cn

**Keywords:** remote sensing image dehazing, unsupervised generating adversarial network, multi-scale feature-extraction module, attention module

## Abstract

The degradation of visual quality in remote sensing images caused by haze presents significant challenges in interpreting and extracting essential information. To effectively mitigate the impact of haze on image quality, we propose an unsupervised generative adversarial network specifically designed for remote sensing image dehazing. This network includes two generators with identical structures and two discriminators with identical structures. One generator is focused on image dehazing, while the other generates images with added haze. The two discriminators are responsible for distinguishing whether an image is real or generated. The generator, employing an encoder–decoder architecture, is designed based on the proposed multi-scale feature-extraction modules and attention modules. The proposed multi-scale feature-extraction module, comprising three distinct branches, aims to extract features with varying receptive fields. Each branch comprises dilated convolutions and attention modules. The proposed attention module includes both channel and spatial attention components. It guides the feature-extraction network to emphasize haze and texture within the remote sensing image. For enhanced generator performance, a multi-scale discriminator is also designed with three branches. Furthermore, an improved loss function is introduced by incorporating color-constancy loss into the conventional loss framework. In comparison to state-of-the-art methods, the proposed approach achieves the highest peak signal-to-noise ratio and structural similarity index metrics. These results convincingly demonstrate the superior performance of the proposed method in effectively removing haze from remote sensing images.

## 1. Introduction

Remote sensing images are captured by remote sensing devices, such as satellites or airplanes, enabling the acquisition of information about specific areas or objects from a distance. These images play a crucial role in obtaining data on land cover and land use patterns [1]. They facilitate the identification of urban growth, deforestation, crop yields, and other changes related to land use. Moreover, remote sensing images provide valuable insights into environmental factors, including air and water quality, soil moisture, and vegetation health [2]. Analyzing these data aids in our comprehension of the impact of human activities on the environment. Additionally, remote sensing images are instrumental in managing natural disasters such as floods, earthquakes, and wildfires. They offer real-time information that assists authorities in making prompt decisions [3]. Furthermore, remote sensing images are also widely used in the fields of geology and geomorphology [4], archaeology, and cultural heritage [5], as well as climate change studies [6] and other fields [7].

The quality of remote sensing images directly impacts their interpretability, the accuracy of information extraction, the effectiveness of change detection, their mapping precision, the reliability of calibration, and their seamless integration with other datasets, as well as their overall utility for decision-making and research [8]. As such, the assurance of high-quality remote sensing images is paramount in acquiring dependable and valuable information about the Earth’s surface. Nevertheless, a challenge encountered by remote sensing systems is the existence of atmospheric haze. As light traverses through the atmospheric haze, it interacts with minuscule particles suspended in the air, leading to scattering in multiple directions and deviation from its initial trajectory. Consequently, the remote sensing sensor captures scattered light instead of the direct signal, leading to a degradation in image clarity, color accuracy, and detail.

To alleviate the effects of haze on remote sensing images and enhance their quality, numerous methods have been proposed in recent years. These methods can be categorized into two primary categories: a priori-based methods and learning-based methods. A priori-based methods for remote sensing image dehazing rely on prior knowledge or assumptions about the scene and the haze to estimate and remove atmospheric degradation. These methods are capable of accurately reducing haze and restoring the true colors and details of the scene [9]. However, a priori-based methods heavily rely on assumptions about the scene and haze properties. If these assumptions are not valid or are inaccurate, the dehazing performance can be compromised [10]. For example, incorrect estimations of the atmospheric model or scattering properties may lead to inadequate haze removal or the introduction of artifacts in the image. Moreover, a priori-based methods are formulated on the foundation of distinct assumptions and models, which might not possess universal relevance across all situations. Although they could exhibit a strong performance under particular circumstances, their effectiveness might be constrained when faced with varying environmental conditions or diverse atmospheric compositions. This lack of adaptability curtails the applicability of these methods, confining them to specific contextual constraints.

Learning-based methods for remote sensing image dehazing employ deep learning algorithms to learn the mapping between hazy and haze-free images [11]. These methods utilize deep convolutional networks that are trained using a large dataset of paired hazy and haze-free images. By leveraging this dataset, the trained network can automatically discover complex patterns and relationships between the input hazy images and their corresponding haze-free counterparts. Consequently, learning-based methods exhibit more adaptive and robust dehazing performances. One notable advantage of learning-based methods is their potential for generalization across different hazy conditions, environmental settings, and atmospheric compositions. By training deep convolutional networks on diverse datasets, these methods can learn to handle a wide range of haze characteristics and effectively remove haze from various remote sensing images. This generalization capability proves particularly valuable in real-world scenarios where atmospheric conditions may vary. A special type of deep learning method used in remote sensing image dehazing is the generative adversarial network (GAN). Comprising a generative network and an adversarial network, the GAN has shown superior performance compared to conventional deep learning methods [12]. As a result, the GAN framework is also utilized in the field of remote sensing image dehazing.

Although haze-removal methods for remote sensing images based on generative adversarial networks exhibit better dehazing effects compared to other techniques, dehazed images still exhibit a notable color distortion and loss of details. To address this issue, we have introduced an unsupervised generative adversarial network designed to mitigate haze in remote sensing images. It comprises two components: two generative networks with identical structures and two discriminative networks with identical structures. One of the generative networks is responsible for removing haze from remote sensing images, while the other is designed to generate remote sensing images with haze. The discriminative networks are utilized to determine whether an image is generated or real.

The main contributions of this paper are summarized below:

1. We introduce a novel approach by proposing a multi-scale feature-extraction module that enables the extraction of features with different receptive fields. These modules are utilized to design the generative network with an encoder–decoder structure. To mitigate information loss, skip connections, proposed attention modules, and channel concatenation operations are employed to fuse the extracted features in both the encoder and decoder networks.
2. We present a multi-scale adversarial network that enhances the performance of the generative network. This is achieved by designing three parallel convolutional branches with different receptive fields to extract features. The proposed attention module is also utilized to focus the network on important information.
3. We propose an improved loss function for the designed generative adversarial network. This is achieved by incorporating the color constancy loss into the conventional loss function, thereby enhancing the ability of the network to preserve color information.

## 2. Related Work

Numerous image-dehazing methods have been proposed in recent years. These methods can be broadly classified into two categories: a priori-based methods and learning-based methods. A priori-based dehazing methods employ prior information to estimate the global atmospheric and medium transmission map from the input images. One prominent a priori-based method is the DCP (dark channel prior) method proposed by He et al. [13]. This method employs the dark channel prior to removing the haze. In local patches of haze-free images, a significant proportion of the pixels display remarkably low intensities in at least one color channel. By utilizing this prior knowledge in conjunction with the haze imaging model, it becomes possible to directly calculate the thickness of the haze and, consequently, restore a high-quality image. Nonetheless, in situations where scene objects closely resemble the atmospheric light, the method encounters challenges in accurately identifying and mitigating the presence of haze. Another a priori-based method is the LSP (low-rank and sparse prior) method proposed by Bi et al. [14]. This approach utilizes an atmospheric scattering model to decompose the dark channel of a hazy image into two components: the dark channel of direct attenuation with sparseness and the atmospheric veil with low rank. By reformulating the dehazing challenge as a low-order sparse decomposition problem, this approach improves the resilience to prior assumptions and adeptly retrieves depth-related details. However, it is worth noting that this technique might introduce artifacts when applied to real-world images. Berman et al. proposed an algorithm based on a non-local prior that utilizes haze-lines [15]. By using these haze-lines, the algorithm successfully recovers the atmospheric light and the haze-free image. However, this method is not applicable in cases of uneven lighting.

With the advancement of deep learning, numerous learning-based methods have emerged and been applied in noise suppression, image enhancement, and image dehazing [16,17]. Bie et al. introduced the GPD-Net (Gaussian and physics-guided dehazing network) method [18], which employs a Gaussian process in the intermediate latent space to aid in the recovery of clear images. Additionally, it incorporates physical prior information to refine the dehazing results. While this method can effectively restore clear images, the manual parameter settings significantly influence the outcomes. Li et al. introduced a haze-transfer and feature-aggregation network [19], which employs a haze-transfer network and a feature-aggregation network for dehazing. However, the computational complexity of this method is a drawback. Susladkar et al. developed the ClarifyNet method [20], an end-to-end network for single-image dehazing. This model is adept at recovering a significant portion of intricate details, although it might not consistently achieve accurate restoration of object outlines. In the work of Lin et al., the multiscale attention feature fusion network method was introduced [21]. This method was groundbreaking for directly integrating the attention mechanism, as opposed to embedding it within specific modules, for the task of single-image dehazing. The model achieves satisfactory results in image dehazing, but it may introduce color distortion. Fan et al. proposed the multiscale cross-connected dehazing network model [22], which encodes and decodes the hazy image and depth image separately. It incorporates cross-connections at the decoding stage to generate a clean image in an end-to-end manner. Nevertheless, the restoration of color in the dehazed image might not be optimal, especially when dealing with nonhomogeneous haze scenarios.

The application of generative adversarial networks [23] has demonstrated better performances such as with image super-resolution [24], image fusion [25], and image dehazing. Indeed, researchers have also employed generative adversarial networks to address the challenge of image dehazing. Park et al. proposed a heterogeneous generative adversarial network (GAN) [26], which combines a cycle-consistent generative adversarial network (CycleGAN) [27] and a conditional generative adversarial network (cGAN) [28]. While this network is capable of restoring clear images from heavily hazy ones, it is important to note that the contrast ratio of the dehazed images might not always reach an optimal level. Zhu et al. introduced a novel generative adversarial network (GAN) for single-image dehazing [29]. Their method employs an adversarial combinatorial network to learn the physical parameters and recover clean images from blurred images in an end-to-end manner. This approach achieves good results in terms of the colors and details in dehazed images but does not effectively remove haze from the sky area. Dong et al. proposed the FD-GAN (generative adversarial networks with fusion discriminator) method [30], which incorporates frequency information as additional priors. This network excels in generating dehazed images that appear more natural and realistic, displaying fewer artifacts. However, it is worth mentioning that there is a possibility of slight color distortion being introduced in the process. Li et al. developed a novel single-image dehazing algorithm that combines model-based and data-driven approaches [31]. The algorithm first utilizes a model-based approach to estimate the global atmospheric conditions and the medium transmission map and then employs a dual-scale generative adversarial network approach to dehaze the image. While this network demonstrates a strong performance in terms of its dehazing effects, it encounters certain limitations when handling images with intense haze. Mo et al. proposed the DCA-CycleGAN (dark channel attention-optimized CycleGAN) method [32], designed to address challenging scenes with uneven and dense haze concentrations. This network takes dark-channel images as input and utilizes the DCA sub-network to handle nonhomogeneous haze. It incorporates both a traditional global discriminator and two local discriminators to facilitate the dehazing process. The network achieves good dehazing results on outdoor datasets, but the performance on indoor datasets is less satisfactory. Zheng et al. introduced the Dehaze-AGGAN (dehazing using enhanced attention-guide generative adversarial networks) method [33], which utilizes an enhanced attention module to guide the training of the generative adversarial network. The method achieves favorable outcomes in terms of generating dehazed images; however, it might fall short of completely recovering intricate details. Chen et al. proposed the MO-GAN (memory-oriented generative adversarial network) method [34], which aims to capture desired hazy features in an unpaired learning manner specifically for single remote sensing image dehazing (RSID). However, the network does not completely remove the haze. Wang et al. introduced the TMS-GAN (twofold multi-Scale generative adversarial network) architecture [35]. This approach involves employing a haze-generation GAN to synthesize hazy images from real-world images, followed by utilizing a haze-removal GAN network for the dehazing process. Ren et al. proposed an unsupervised dehazing algorithm based on a GAN [36], which employs two discriminators to consider global and local information along with a dark-channel attention mechanism. While most of the haze can be removed using this approach, some residual haze may remain.

Generative adversarial networks have shown promising outcomes in image dehazing. Nonetheless, the dehazed images frequently experience a color distortion and a loss of intricate details, greatly affecting the output’s quality. To address the restoration of detailed information in dehazed remote sensing images and mitigate color loss, we introduce an unsupervised generative adversarial network. This network incorporates a multi-scale feature-extraction module and introduces a color-constancy loss specifically designed for remote sensing image dehazing.

## 3. Unsupervised Generative Adversarial Network for Remote Sensing Image Dehazing

**Figure 1:**
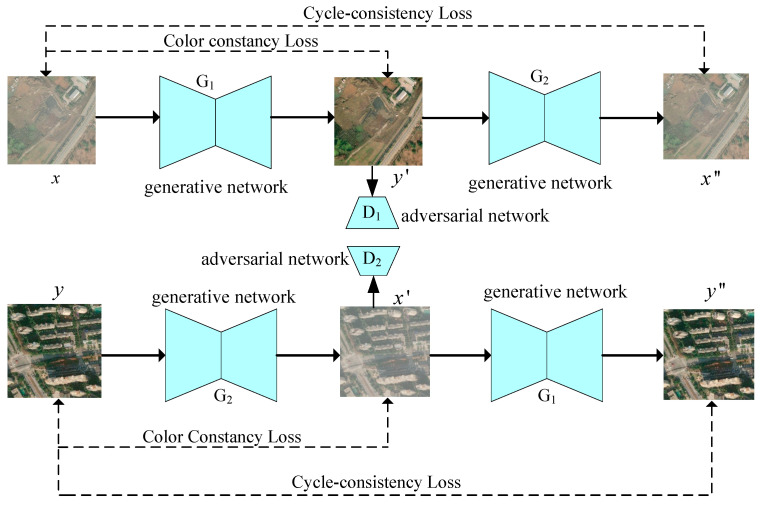
Network framework.

To reduce the impact of haze on remote sensing image quality, we proposed an unsupervised generative adversarial network (GAN) that can be trained using unpaired remote sensing data. Our network framework is based on the cycle-GAN architecture, as illustrated in Figure 1. This network framework contains two generative networks (G1 and G2) and two adversarial networks (D1 and D2). The generative network G1 is responsible for recovering the hazy remote sensing image to a clear image. The adversarial network D1 is responsible for distinguishing whether the image generated by the generator G1 is real or reconstructed. Similarly, the generative network G2 generates a hazy image from the high-quality remote sensing image. The adversarial networkD2 distinguishes whether the image generated by the generator G2 is a real image or a generated image. It can improve the ability of the generative network to generate images and the ability of the adversarial network to judge image authenticity. G1 and G2 have the same network structure. D1 and D2 also have the same network structure. They share weights during training. In Figure 1, x and y are the hazy remote sensing image and clear remote sensing image, respectively. The cycle-consistency loss is employed to quantify the difference between the input image and the image generated by two consecutive adversarial networks. The color-constancy loss is utilized to measure the color difference between the input image and the image generated by the adversarial network. In the following sections, we will introduce how to design the generative network and adversarial network.

### 3.1. Proposed Generative Network

The generative network we propose incorporates our novel attention modules and multi-scale feature extraction modules. As a result, we will begin by providing individual introductions to these designed modules. Subsequently, we will elaborate on how these modules can be effectively employed to establish the framework of the new generative network.

#### 3.1.1. Proposed Attention Module

**Figure 2:**
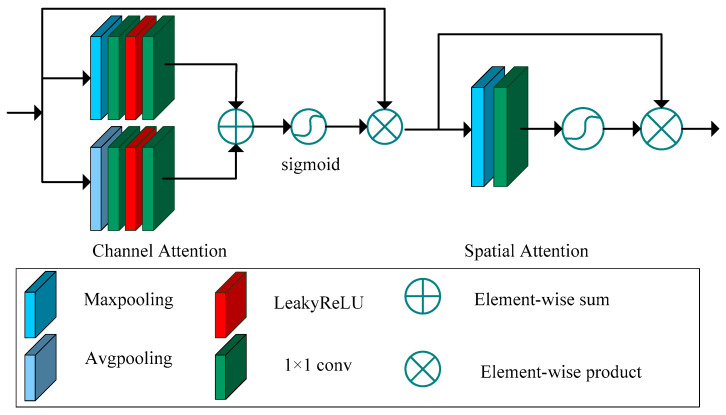
Our proposed attention module.

The attention module proposed for use in the generative network, as well as the multi-scale feature-extraction module, is illustrated in Figure 2. The attention module guides the feature-extraction network to focus more on the haze and texture of the remote sensing image. This mechanism effectively enhances the overall quality of the remote sensing image. The attention mechanism consists of a channel attention module and a spatial attention module.

The channel attention module enhances feature representation in the channel dimension. This module comprises two distinct branches. The first branch includes maximum pooling, a 1 × 1 convolution, a LeakyReLU activation function, and another 1 × 1 convolution. The second branch comprises average pooling, a 1 × 1 convolution, a LeakyReLU activation function, and another 1×1 convolution. The maximum pooling and average pooling operations are employed to extract texture information and background feature information, respectively. The 1 × 1 convolutions in both branches adjust the number of channels to enhance inter-channel correlation. The extracted features are fused using an element-wise sum operation. Finally, a sigmoid function is employed to derive the channel attention weights from the fused features. These computed weights are then multiplied with the input feature map, resulting in a new feature map. This new feature map serves as the input for the subsequent spatial attention module.

The output feature map of the channel attention module can be expressed as follows:

(1) $$F_{1}(x)=x\cdot \sigma \{[Conv_{1}(LReLU(Conv_{2}(avg(x))))]⊕[Conv_{1}(LReLU(Conv_{2}(max(x))))]\}$$

where x denotes the denotes the input feature map of the channel attention. σ(⋅) denotes the sigmoid activation function. Conv(⋅) denotes 1 × 1 convolution. LReLU denotes the LeakyReLUactivation function. avg(⋅) denotes average pooling. max(⋅) denotes average pooling.

The spatial attention module is used to improve the feature representation in the spatial dimension. The spatial attention module consists of a maximum pooling layer, a 1 × 1 convolution, and a sigmoid function. In the spatial attention module, maximum pooling is used to extract texture information in the spatial dimension. In the end, the sigmoid function is used to obtain the spatial feature weights. The output of the spatial attention module can be expressed as:

(2) $$F_{2}(x)=x\cdot \sigma [Conv(max(x))]$$

where x denotes the input feature map of the spatial attention. σ(⋅) denotes the sigmoid activation function. Conv(⋅) denotes 1 × 1 convolution. max(⋅) denotes average pooling.

In the designed attention module, the channel attention module and spatial attention module collaborate to assign larger weights to significant features in the channel dimension and spatial dimension, respectively.

#### 3.1.2. Proposed Multi-Scale Feature-Extraction Module

**Figure 3:**
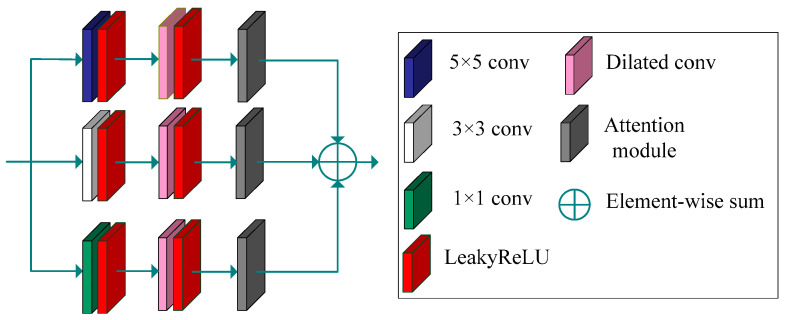
Multi-scale feature-extraction module.

In the proposed generative network, a multi-scale module incorporates a designed attention module. This combination aims to extract more effective information from different receptive fields. Therefore, we first introduce our designed multi-scale module and attention module. The multi-scale module, shown in Figure 3, is employed to extract features from different scales. We utilize convolution with different kernel sizes to extract depth information. This module comprises three branches.

The first branch consists of a 5 × 5 convolution layer with a LeakyReLU activation function, a dilated convolution with a LeakyReLU activation function, and our designed attention module. The second branch consists of a 3 × 3 convolution layer with a LeakyReLU activation function, a dilated convolution with a LeakyReLU activation function, and our designed attention module. The third branch consists of a 1 × 1 convolution layer with a LeakyReLU activation function, a dilated convolution with a LeakyReLU activation function, and our designed attention module. The purpose of the dilated convolution is to expand the receptive fields and extract more lower-frequency information. The dilation rates of the dilated convolutions are 5, 3, and 1 for the three branches, respectively.

The conventional convolution utilized within the multi-scale module serves a dual purpose: not only does it facilitate feature extraction, but it also mitigates the tessellation effect arising from the application of dilated convolutions. The attention module enables the network to focus on the important feature information and extract more effective features. Finally, the features extracted from the three branches are fused using an element-wise sum operation.

#### 3.1.3. Complete Generative Network

**Figure 4:**
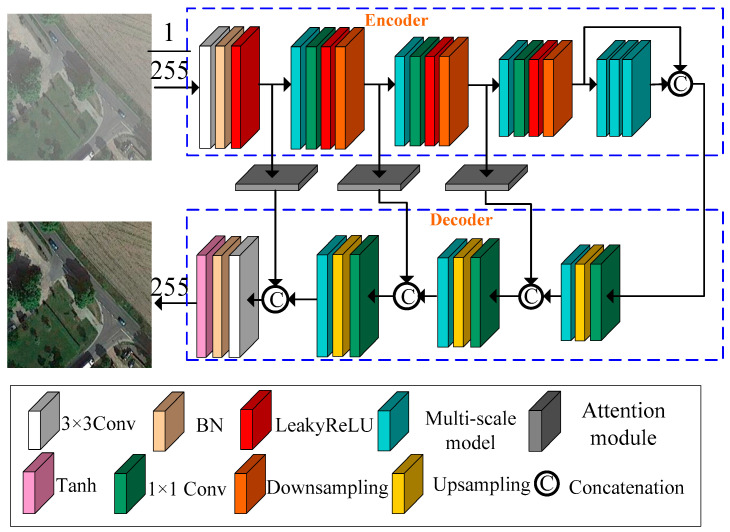
Proposed generator.

The depicted generative network with an encoder–decoder structure is presented in Figure 4. It predominantly comprises an encoder and a decoder. In the encoder part, we extract the shallow features of the remote sensing image by increasing the number of channels and reducing the feature map size. In the decoder part, we extract deeper features of the remote sensing image by decreasing the number of channels and increasing the feature map size. Finally, we employ a 3 × 3 convolutional layer with batch normalization and a Tanh activation function to reconstruct the remote sensing image from the extracted features.

In the encoder part, we first apply a 3 × 3 convolution layer with batch normalization and a LeakyReLU activation function to increase the number of channels from 3 to 32. Next, we employ the proposed multi-scale module shown in Figure 3, followed by a 1 × 1 convolution with a LeakyReLU activation function and a downsampling operation to create a module group. We repeat this process three times to further extract features. The 1 × 1 convolution with a LeakyReLU activation function enhances network nonlinearity, while the downsampling operation reduces the size of the input feature map by half and doubles the number of channels. Consequently, the output feature maps for the three module groups are 1/2, 1/4, and 1/8 of the size of the input feature map, with respective channel numbers of 64, 128, and 256. Finally, three multi-scale modules are employed without altering the size of the feature map to increase the depth of the network and extract more complex feature information. The output feature map of the encoder is then used as the input feature map for the decoder.

In the decoder part, we utilize a combination of 1 × 1 convolutions, upsampling operations, and our proposed multi-scale module shown in Figure 4 to create a module group. The 1 × 1 convolution is employed to merge information from different channels, while the upsampling operation increases the size of the feature map and reduces the number of channels. The three module groups have the channel numbers 256, 128, and 64, respectively. The output feature maps for these module groups in the decoder part have sizes of 2, 4, and 8 times the input feature map, respectively.

To minimize the potential loss of feature information, we introduce three shortskip connections integrated with attention modules. This combination serves to merge the features extracted by the module groups within both the encoder and decoder sections. This approach enhances the network’s learning capability by preserving detailed information and capturing more underlying features. Finally, the remote sensing image is reconstructed using a 3 × 3 convolution layer with batch normalization and a Tanh activation function applied to the extracted features.

### 3.2. Proposed Adversarial Network

**Figure 5:**
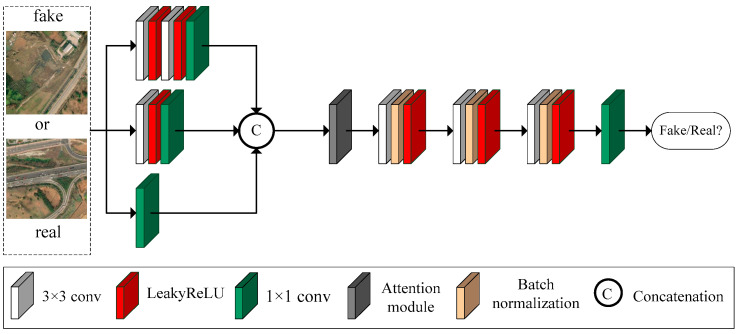
Proposed discriminator.

The generative adversarial network consists of a generative network and an adversarial network. The generative network is designed to remove haze from remote sensing images, while the adversarial network is responsible for determining whether a given remote sensing image is a dehazed image or the original clear image. The adversarial network plays a crucial role in enhancing the performance of the generative network. In this paper, we outline the architecture of the adversarial network, as illustrated in Figure 5.

Firstly, we design a multi-scale module with three branches for feature extraction. The first branch includes two 3 × 3 convolutions with LeakyReLU activation functions and a 1 × 1 convolution. The second branch comprises a 3 × 3 convolution with a LeakyReLU activation function and a 1 × 1 convolution. The third branch consists of a 1 × 1 convolution. These three branches extract features at different scales, with receptive fields of 5 × 5, 3 × 3, and 1 × 1, respectively. The output feature maps of all three branches have 32 channels and a size of 256 × 256. These feature maps are then concatenated to fuse the information.

Moreover, we have designed an attention module, depicted in Figure 2, to aid the adversarial network in directing its attention toward crucial information. Additionally, three 3 × 3 convolutions with batch normalization and LeakyReLU activation functions are utilized to further extract features. Lastly, a 1 × 1 convolution is employed to combine the features and determine whether the input image is a generated image or a real image.

### 3.3. Improved Loss Function

To achieve a more precise assessment of the generative network and adversarial network’s performance, we introduce a novel loss function for our generative adversarial network. This innovative loss function integrates the conventional loss with the color-constancy loss. The complete loss function comprises several components, including adversarial loss, cycle-consistency loss, perceptual loss, color-constancy error, and identity loss. It is defined as follows:

(3) $$L=\lambda_{1}L_{ad}(G)+\lambda_{2}L_{cyc}+\lambda_{3}L_{idt}+\lambda_{4}L_{per}+\lambda_{5}L_{\mathrm{col}}$$

where $L_{ad}()$, $L_{cyc}()$, $L_{idt}()$, $L_{per}()$, and $L_{\mathrm{col}}()$ are adversarial loss, cycle-consistency loss, identity loss, perceptual loss, and color-constancy loss, respectively. The weights of each loss function, in turn, are 0.1, 0.1, 1, 0.05, and 0.5 in (3), respectively. The adversarial loss is expressed as follows:

(4) $$L_{ad}(G)=E[Sm(D_{1}(y)-1)]+E[Sm(D_{1}(y’))]+E[Sm(D_{2}(x)-1)]+E[Sm(D_{2}(x’))]$$

where x is the original remote sensing image with haze, y is the original remote sensing image without haze, x’ is the hazed image generated by generator $G_{2}$, y’ is the dehazed image generated by generator $G_{1}$, and the Sm() is the Smooth L1 Loss that is expressed as:

(5) $$Sm(x)=\left\{\begin{array}{c} \begin{array}{cc} 0.5x^{2} & \left|x\right|<1 \end{array}\\ \begin{array}{cc} \left|x\right|-0.5 & \left|x\right|>1 \end{array} \end{array}\right.$$

The cycle-consistency loss is expressed as follows:

(6) $$L_{cyc}=E[Sm(G_{2}(G_{1}(x))-x)]+E[Sm(G_{1}(G_{2}(y))-y)]$$

where $G_{2}(G_{1}(x))$ is the generated remote sensing image with haze (blurred images) from the generated dehazed remote sensing image by $G_{1}(x)$, and $G_{1}(G_{2}(x))$ is the generated dehazed remote sensing image from the generated remote sensing image with haze (blurred images) by $G_{2}(x)$. The identity loss is expressed as follows:

(7) $$L_{idt}=E[Sm(G_{1}(y)-y)]+E[Sm(G_{2}(x)-x)]$$

The perceptual loss is expressed as follows:

(8) $$L_{per}=\sum_{l=3}^{5}\left\{Sm[\Phi_{l}(G_{1}(x))-\Phi_{l}(x)]+Sm[\Phi_{l}(G_{2}(y))-\Phi_{l}(y)\right]\}$$

where $\Phi_{l}(\cdot )$ is the extracted feature map. l indicates the third, fourth, and fifth layers in the VGG-16 network. The color-constancy loss is expressed as follows:

(9) $$L_{\mathrm{color}}=\sum_{\forall (p,q)\in \omega }{\left(J^{p}-J^{q}\right)}^{2},\omega =\{(R,G),(R,B),(B,G)\}$$

where $J^{p}$ denotes the average intensity value of the *p* channel in the dehazed image. (p,q) represents a paired channel.

## 4. Simulation and Discussion

To evaluate the effectiveness of our proposed method, we conducted simulation experiments using the RESISC45 dataset [37]. The RESISC45 dataset consists of 31,500 clear remote sensing images captured from 45 different scenes, with each image having a size of 256 × 256 pixels. For our experiments, we randomly selected 3500 images from the RESISC45 dataset to synthesize hazy remote sensing images using an atmospheric scattering model. These synthesized images were used as the RESISC45test set. The remaining 28,000 images were divided into two parts, with 14,000 images in each part. In one part, we used the images to synthesize hazy remote sensing images using the atmospheric scattering model. In the other part, we combined the images with the synthesized hazy images from the first part to create an unpaired training set. In addition, we also test the dehazing effect of different methods on the LHID dataset [38] and the real remote sensing hazy dataset [39]. The real remote sensing hazy data set consists of 150 real outdoor hazy images collected by an unmanned aerial vehicle for remote sensing image dehazing. The atmospheric scattering model used in our experiments is defined as follows:

(10) I(x)=J(x)t(x)+A(1−t(x))

where I(x) is the hazy remote sensing image, J(x) is the clear remote sensing image, A is the atmospheric value, and t(x) is the transmittance, and it can be expressed as:

(11) $$t(x)=e^{-\lambda d(x)}$$

where λ is the scattering factor of atmospheric light, which we set randomly to the interval [0.04, 0.1]. d(x) is the scene depth information of the remote sensing image.

We use two evaluation indexes, *PSNR* [40] and *SSIM* [41], to quantitatively compare our method with other methods. The *PSNR* can be expressed as:

(12) $$PSNR=20\log_{10}(\frac{MAX_{I}}{\sqrt{MSE}})$$

where $MAX_{I}$ is the maximum value of image pixels, and *MSE* is the mean square error. The *MSE* can be expressed as:

(13) $$MSE=\sqrt{\frac{1}{wh}\sum_{i=0}^{w-1}{\sum_{j=0}^{h-1}\left||I_{pre}(i,j)-I_{gt}(i,j)|\right|}^{2}}$$

where w and h are the width and height of the image, and $I_{pre}$ and $I_{gt}$ represent the dehazed image and the haze-free image, respectively. The *SSIM* can be expressed as:

(14) $$SSIM(x,y)=\frac{\left(2\mu_{x}\mu_{y}+c_{1})(2\sigma_{xy}+c_{2}\right)}{\left(\mu_{x}{}^{2}+\mu_{y}{}^{2}+c_{1})(\sigma_{x}{}^{2}+\sigma_{y}{}^{2}+c_{2}\right)}$$

where $\mu_{x}$ and $\mu_{y}$ are the mean values of x and y, $\sigma_{x}{}^{2}$ and $\sigma_{y}{}^{2}$ are the variances of x and y, and $\sigma_{xy}$ is the covariance of x and y.

In our experiment, the network was trained with 200 epochs at a learning rate of 2 × 10^−4^ for the first 100 epochs and a linear decrease to 0 for the next 100 epochs. We used the Adam optimizer to optimize the network, and parameters $\beta_{1}$, $\beta_{2}$ were set to 0.9 and 0.999, respectively. The whole training process is described in Algorithm 1. We used Ubuntu 18.04 system in our experiment. The GPU is the NVIDIA GeForce GTX 1080ti, and the deep learning framework is PyTorch.
**Algorithm 1:** Training procedure for our proposed method.1: **For**
*K* epochs **do** 2:     **For**
*k*(*k* is a hyperparameter, *k* = 1) steps **do** 3:     Sample minibatch of m hazy image samples {*z*^(1)^, …, *z*^(m)^} from hazy image domain. 4:     Sample minibatch of m haze-free image samples {*z*^(1)^, …, *z*^(m)^} from haze-free image domain. 5:     Update the discriminator by Adam optimizer:      $\nabla_{D}\left\{E{\left[\left(D(x^{\left(i\right)})-1\right)\right]}^{2}+E{\left[\left(D(z^{\left(i\right)})\right)\right]}^{2}\right\}$ 6:    **End for** 7:    Sample minibatch of m hazy image samples {z^(1)^, …, z^(m)^} from hazy image domain. 8:    Update the generator by Adam optimizer:      $\nabla_{D}\left\{E{\left[\left(D(G(z^{\left(i\right)}))\right)\right]}^{2}\right\}$ 9: **End for**

### 4.1. Simulation on the RESISC45 Dataset

**Figure 6:**
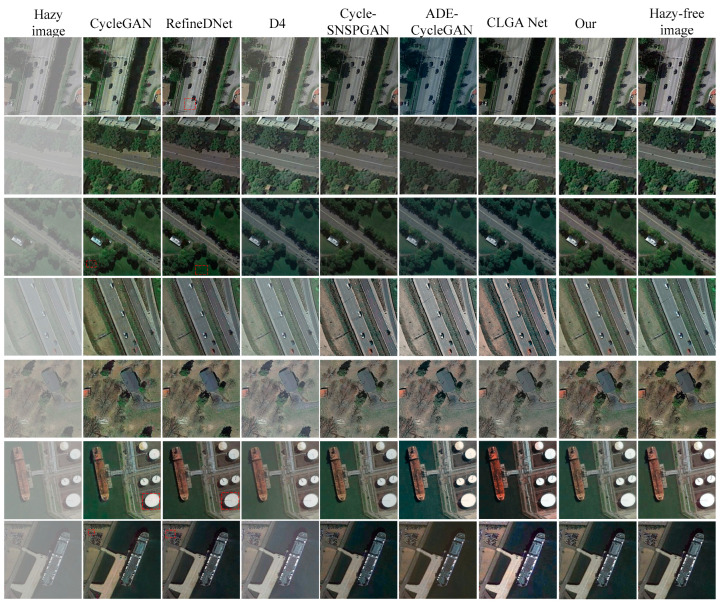
Dehazing results of different methods on synthesized hazy remote sensing images.

We randomly selected seven hazy remote sensing images from the RESISC45test set to compare the dehazing performance of our proposed method with the CycleGAN method [27], the RefineDNet method [42], the Cycle-SNSPGAN method [43], the D4 method [44], the ADE-CycleGAN method [45], and the CLGA Net method [46]. The images are displayed in Figure 6, showcasing the hazy images, dehazed images using different methods, and haze-free images. The first to ninth columns correspond to the input hazy remote sensing images, the dehazed images generated by the CycleGAN method, the RefineDNet method, the D4 method, the Cycle-SNSPGAN method, the ADE-CycleGAN method, the CLGA Net method, our proposed method, and the haze-free remote sensing images, respectively.

In the first row, we observe that the CycleGAN method successfully removes haze from the hazy image, but it introduces color distortion. The dehazed image generated by the RefineDNet method fails to effectively restore the detailed information of the original remote sensing images (marked in red). The D4 method retains some amount of haze in the dehazed images. The dehazed image obtained from the Cycle-SNSPGAN method is over-enhanced. The dehazed image generated by the ADE-CycleGAN method fails to remove the haze. The dehazed image obtained from the CLGA Net method exhibits color distortion. However, our proposed method produces a more natural-looking dehazed image compared to the other methods.

In the second row, the dehazed image generated by the CycleGAN method exhibits color distortion. The dehazed image obtained from the RefineDNet method is over-enhanced, resulting in a darkened brightness. Both the Cycle-SNSPGAN method and the D4 method still struggle to effectively remove the haze. The dehazed image generated by the ADE-CycleGAN method fails to remove haziness. The dehazed image obtained from the CLGA Net method exhibits color distortion. However, our proposed method generated a dehazed image that is clearer and closer to the haze-free reference image compared to the other methods.

In the third row, the dehazed image generated by the CycleGAN method loses a significant amount of detailed information. The RefineDNet method introduces artifacts (marked in red) in its dehazed image. The D4 method fails to adequately remove the haze, leaving a considerable amount in the resulting image. The dehazed image produced by the Cycle-SNSPGAN method appears to be excessively enhanced. The dehazed images generated by the ADE-CycleGAN method and the CLGA Net method exhibit color distortion. In contrast, our proposed method achieves better results, with a dehazed image that closely resembles the haze-free reference.

In the fourth and fifth rows, the dehazed images obtained through the CycleGAN method exhibit a loss of detail. The D4 method continues to struggle with haze removal. The dehazed images produced by the Cycle-SNSPGAN method show color distortion, while the RefineDNet method fails to effectively restore color information. Conversely, our proposed method proves effective in removing haze and yields dehazed images that closely resemble the haze-free reference. In the sixth and seventh rows, both the CycleGAN and RefineDNet methods result in a further loss of detailed information in the dehazed images. The D4 method still falls short in removing the haze. The dehazed image generated by the ADE-CycleGAN method shows color distortion. The dehazed image obtained through the CLGA Net method is overexposed.

In summary, the dehazed images generated by the CycleGAN method suffer from a loss of detailed information. While the RefineDNet method proves effective in removing haze from images, the color in the dehazed images produced by this method appears to be over-enhanced. The D4 method fails to effectively remove haze from the remote sensing images. Additionally, the dehazed images obtained through the Cycle-SNSPGAN method exhibit color distortion. The dehazed images generated by the ADE-CycleGAN method and the CLGA Net method also exhibit color distortion. Comparatively, our proposed method generated dehazed images that are the clearest, and the color is closest to that of the haze-free remote sensing images when compared to the other four methods.

To quantitatively analyze the performance of different methods, we employed PSNR and SSIM as evaluation metrics to compare the dehazing performance of the CycleGAN method, RefineDNet method, D4 method, Cycle-SNSPGAN method, ADE-CycleGAN method, CLGA Net method, and our proposed method. The test results are presented in Table 1. The PSNR values for the CycleGAN method, RefineDNet method, D4 method, Cycle-SNSPGAN method, ADE-CycleGAN method, CLGA Net method, and our proposed method are 25.178, 27.644, 25.786, 28.667, 28.674, 28.934, and 29.885, respectively. In terms of SSIM, the CycleGAN method, RefineDNet method, D4 method, Cycle-SNSPGAN method, ADE-CycleGAN method, CLGA Net method, and our method achieved scores of 0.839, 0.894, 0.867, 0.954, 0.952, 0.956, and 0.964, respectively. Notably, our proposed method demonstrated the highest PSNR and SSIM values, indicating superior dehazing performance compared to the other methods.

### 4.2. Simulation on Remote Sensing Images with Different Haze Thicknesses

**Figure 7:**
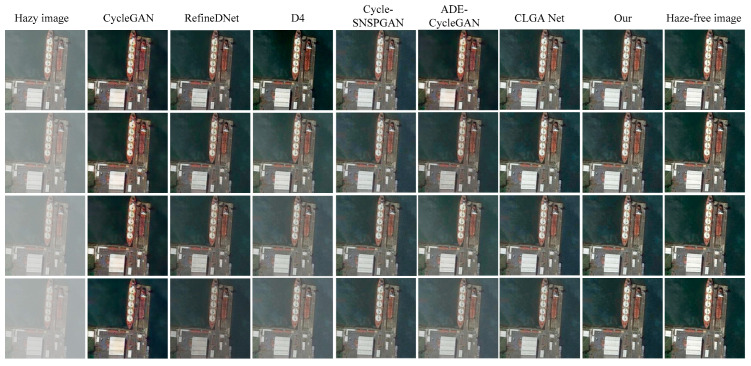
Images with different haze thickness and dehazed images by different methods.

To assess the image dehazing performance of each method at varying haze thicknesses, we randomly selected an image from the RESISC45 dataset and adjusted the scattering factor to 0.04, 0.06, 0.08, and 0.1, effectively altering the levels of haze thickness according to an atmospheric scattering model. Figure 7 showcases the dehazed images generated by different methods at these different haze thicknesses. Observing the results, it becomes evident that as the haze thickness increases, the dehazing performances of the various methods deteriorate. Nonetheless, when comparing images with the same haze thickness, our method restores detailed information in the dehazed image and provides superior color representation.

Table 2 displays the PSNR and SSIM values of the different methods. The results indicate that our proposed method consistently achieves the highest PSNR and SSIM values for images with the same haze thickness. This shows that our method is better-suited for the task of remote sensing image dehazing.

### 4.3. Simulation on the LHID Dataset

**Figure 8:**
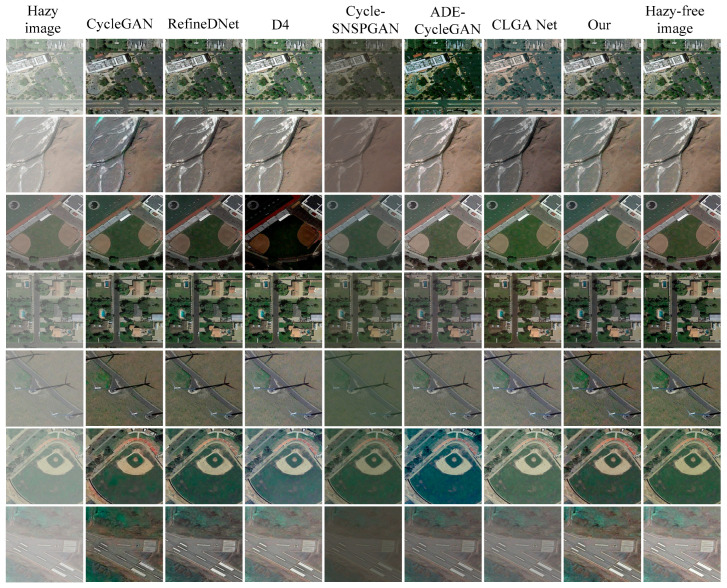
Dehazing results of different methods on the LHID dataset.

We randomly selected seven images from the LHID dataset to evaluate the image dehazing performance of different methods. The dehazed images produced by each method are presented in Figure 8. The first column displays the original hazy remote sensing images, while the second to ninth columns depict the dehazed images generated by the CycleGAN method, the RefineDNet method, the D4 method, the Cycle-SNSPGAN method, the ADE-CycleGAN method, the CLGA Net method, our proposed method, and the haze-free remote sensing images, respectively.

In the first row, the dehazed image generated by the CycleGAN method fails to effectively restore the detailed information of the original remote sensing images. The dehazed images obtained from the RefineDNet method and the CLGA Net method exhibit color distortion. The dehazed images obtained from the D4 and ADE-CycleGAN methods are over-enhanced. The dehazed image obtained from the Cycle-SNSPGAN method fails to remove the haze and exhibits color distortion. However, our proposed method produces a more natural-looking dehazed image compared to the other methods.

In the second row, the dehazed images generated by the CycleGAN method, the RefineDNet method, and the CLGA Net method exhibit color distortion. The dehazed image obtained from the Cycle-SNSPGAN method fails to remove haziness and exhibits color distortion. In the third row, the dehazed image obtained from the RefineDNet method exhibits color distortion. The dehazed image obtained from the D4 method exhibits severe color distortion. The Cycle-SNSPGAN method and the ADE-CycleGAN method retain an amount of haze in the dehazed images. In the fourth row, the ADE-CycleGAN method still retains some amount of haze in the dehazed images. In contrast, our proposed method achieves better results, with a dehazed image that closely resembles the haze-free reference.

In the fifth and sixth rows, the dehazed images obtained through the CycleGAN method exhibit a loss of detail. The D4 method, the ADE-CycleGAN method, and the CLGA Net method continue to struggle with haze removal. The dehazed images produced by the Cycle-SNSPGAN method show color distortion. In the seventh row, the CycleGAN method results in a further loss of detailed information in the dehazed images. Images obtained from the D4 method and the CLGA Net method still contain a significant amount of haze residue. The dehazed images generated by the Cycle-SNSPGAN method and the ADE-CycleGAN method exhibit color distortion.

In summary, the dehazed images generated by the CycleGAN method suffer from a loss of detailed information. The dehazed images obtained from the RefineDNet method exhibit color distortion. The color in the dehazed images produced by the D4 method and the ADE-CycleGAN method appears to be over-enhanced. Additionally, the dehazed images obtained through the Cycle-SNSPGAN method exhibit color distortion. The dehazed images generated by the CLGA Net method exhibit color distortion. Comparatively, our proposed method generates dehazed images that are the clearest, and the color is closest to that of the haze-free remote sensing images when compared to the other four methods.

To quantitatively analyze the performance of the different methods, we employed PSNR and SSIM as evaluation metrics to compare the dehazing performance of the different methods. The test results are presented in Table 3. The results indicate that our proposed method has the highest PSNR and SSIM values, demonstrating a superior dehazing performance compared to the other methods.

### 4.4. Simulation on Real Remote Sensing Images

**Figure 9:**
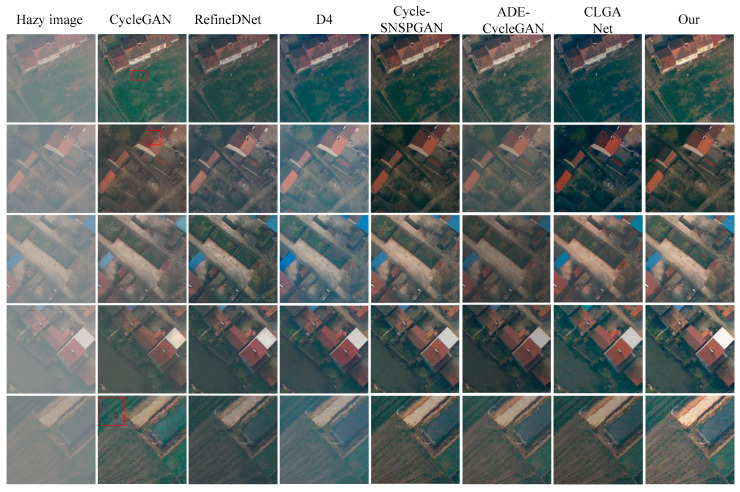
Dehazing results of different methods for real hazy images.

We conducted tests using real hazy remote sensing images to evaluate the image dehazing performance of the different methods. The dehazed images produced by each method are presented in Figure 9. The first column displays the original hazy remote sensing images, while the second to eighth columns depict the dehazed images generated by the CycleGAN method, the RefineDNet method, the D4 method, the Cycle-SNSPGAN method, the ADE-CycleGAN method, the CLGA Net method, and our proposed method, respectively. In the first and second rows, it is evident that the dehazed images obtained through the CycleGAN method fail to restore the original image’s detailed information (highlighted in red). The images generated by the RefineDNet method exhibit over-enhanced colors. The dehazed images generated by the D4 method are unable to effectively remove haze from the real remote sensing images. Furthermore, the dehazed images generated by the Cycle-SNSPGAN method suffer from color distortion. The images generated by the CLGA Net method exhibit color distortion.

In the third and fourth rows, the dehazed images generated by the CycleGAN method exhibit a loss of edge information. The dehazed images produced by the RefineDNet method also display color distortion. The D4 method fails to adequately remove the haze from the images. The images generated by the Cycle-SNSPGAN method exhibit over-enhanced colors. The images generated by the ADE-CycleGAN method exhibit color distortion. In the fifth row, the images obtained through the CycleGAN method exhibit artifact phenomena (marked in red). The dehazed images generated by the RefineDNet method again demonstrate color distortion. The D4 method still struggles to remove the haze effectively. Similarly, the dehazed images generated by the Cycle-SNSPGAN method exhibit color distortion. The dehazed images generated by the ADE-CycleGAN method and the CLGA Net method exhibit color distortion. In summary, the dehazed images generated by our proposed method are clearer and retain more detailed information compared to the other methods.

### 4.5. Ablation Experiments

To evaluate the performance of each module in our proposed method, we conducted ablation experiments. Four experiments were performed on synthesized hazy remote sensing images: one without the color-constancy loss (No_color), one without the multiscale module (No_multi), one without the attention module (No_attention), and one without the multiscale discriminative network (No_msdn). The assessment results for each module are presented in Table 4.

For the No_color experiment, the PSNR result was 28.844, while the SSIM result was 0.932. In the No_multi experiment, the PSNR result was 28.967, and the SSIM result was 0.944. In the No_attention experiment, the PSNR result was 28.378, and the SSIM result was 0.945. In the No_msdn experiment, the PSNR result was 27.365, and the SSIM result was 0.904. Finally, for our proposed method, the PSNR result was 29.885, and the SSIM result was 0.964.

The results clearly demonstrate the significance of the modules proposed in our method during the dehazing process. Each module plays a crucial role in enhancing the performance of the dehazing algorithm.

## 5. Discussion of the Study for SDGs

Remote sensing image dehazing technology can play a significant role in monitoring and managing oceans and marine resources. Clear remote sensing images can assist in monitoring ocean pollution, changes in coastal ecosystems, marine biodiversity, and fisheries’ resources. By reducing the impact of haze, more accurate images can be obtained, contributing to the sustainable management of ocean resources. This technology can also be applied to monitor and protect terrestrial ecosystems. Clear remote sensing images can help identify issues such as land degradation, deforestation, and vegetation changes, thereby supporting sustainable land management and ecosystem protection. By providing detailed image information, the state and health of ecosystems can be better monitored and assessed. Therefore, our approach can provide valuable information for the protection and sustainable utilization of oceans and marine resources, as well as the conservation, restoration, and sustainable use of terrestrial ecosystems, thereby supporting the achievement of these sustainable development goals. Through the provision of clear image data, this technology contributes to the improved monitoring and management of natural resources, promoting environmental conservation and sustainable development.

## 6. Conclusions

In this paper, we propose an unsupervised generative adversarial network for remote sensing image dehazing. For the generative network, we have designed a multi-scale feature-extraction module and an attention module. We design a generator with an encoder–decoder structure that incorporates the multi-scale feature-extraction modules, convolutions, attention modules, and skip connections. As for the discriminative network, we have also designed a multi-scale discriminative network, which effectively enhances the discriminative performance of the network. Additionally, we employed Smooth L1 Loss and color-constancy loss to improve the training stability of the network and reduce color distortion in the dehazed images.

To test the haze removal performance, we use the synthesized hazy remote sensing images and real hazy remote sensing images as the input images of different methods, respectively. For the synthesized hazy remote sensing images, our proposed method better retained the color and detailed information of the original remote sensing images than other methods. The dehazed images generated by our proposed method were closest to the haze-free remote sensing images, followed by those ofthe CLGA Net method, the ADE-CycleGAN method, the Cycle-SNSPGAN method, the RefineDNet method, the D4 method, and the CycleGAN method, respectively. To quantitatively analyze the dehazing performance of these different methods, we also employed PSNR and SSIM as measures. Our proposed method exhibited the highest PSNR and SSIM scores on both the RESISC45 dataset and the LHID dataset. On the RESISC45 dataset, our proposed method achieved a 3.2868% improvement in PSNR and a 0.8368% improvement in SSIM when compared to the second-best method, CLGA Net. On the LHID dataset, our proposed method demonstrated a 4.9676% increase in PSNR and a 2.9605% increase in SSIM compared to the second-best method, CLGA Net.

For the quantitative analysis of the dehazing performance across various haze thicknesses, we utilized images with different atmospheric light scattering factors: 0.04, 0.06, 0.08, and 0.1. These factors were applied to the test images. The outcomes of our experiments reveal that our method consistently outperforms other approaches in terms of PSNR and SSIM, even when confronted with identical haze thickness conditions. Additionally, we employed real remote sensing images as test images to evaluate the dehazing performance of different methods. In contrast to other methods, the dehazed images generated by our method preserve more details and color information. This preservation contributes to clearer images and enhanced visual quality.

The complexity of the model has a direct impact on the dehazing speed. In future work, we will consider reducing the complexity of the multi-scale feature-extraction module. By constructing the generation network with lightweight feature-extraction modules, we aim to achieve a balance between dehazing effectiveness and processing speed. Furthermore, we will also explore the potential application of the module for conventional image dehazing scenarios, where collecting paired images is practically unfeasible.

## Figures and Tables

**Table 1:** Comparison of dehazing performance on the RESISC45 dataset.

	CycleGAN	RefineDNet	D4	Cycle- SNSPGAN	ADE- CycleGAN	CLGA NET	Ours
PSNR	25.178	27.644	25.786	28.667	28.674	28.934	29.885
SSIM	0.839	0.894	0.867	0.954	0.952	0.956	0.964

**Table 2:** Dehazing performance comparison for different haze thicknesses.

		CycleGAN	RefineDNet	D4	Cycle- SNSPGAN	ADE- CycleGAN	CLGA NET	Ours
PSNR	0.04	26.100	24.652	23.713	28.367	25.457	28.674	33.139
	0.06	28.199	24.787	20.695	27.998	24.967	28.436	31.673
	0.08	25.872	23.036	20.035	27.356	22.766	27.975	31.326
	0.1	26.939	20.158	19.783	26.673	21.874	27.648	31.219
	Average	26.778	23.158	21.056	27.598	23.766	28.183	31.839
SSIM	0.04	0.889	0.957	0.922	0.966	0.945	0.968	0.971
	0.06	0.897	0.948	0.899	0.952	0.928	0.958	0.964
	0.08	0.890	0.914	0.884	0.923	0.896	0.934	0.954
	0.1	0.892	0.878	0.836	0.893	0.867	0.927	0.952
	Average	0.892	0.924	0.885	0.933	0.909	0.946	0.960

**Table 3:** Comparison of dehazing performance on the LHID dataset.

	CycleGAN	RefineDNet	D4	Cycle- SNSPGAN	ADE- CycleGAN	CLGANET	Ours
PSNR	26.548	28.134	27.426	27.365	28.754	29.934	31.421
SSIM	0.859	0.885	0.882	0.878	0.895	0.912	0.939

**Table 4:** Assessment results for each module.

	No_Color	No_Multi	No_Attention	No_Msdn	Ours
PSNR	28.844	28.967	28.378	27.365	29.885
SSIM	0.932	0.944	0.945	0.904	0.964

## Data Availability

Not applicable.
